# Pregnancy outcomes of Fabry disease in Austria (PROFABIA)-a retrospective cohort-study

**DOI:** 10.1186/s13023-024-03180-3

**Published:** 2024-04-18

**Authors:** Natalja Haninger-Vacariu, Kyra Anastopoulos, Christof Aigner, Raute Sunder-Plassmann, Constantin Gatterer, Markus Ponleitner, Gere Sunder-Plassmann, Alice Schmidt

**Affiliations:** 1 Division of Nephrology and Dialysis, Department of Medicine III, Medical University of Vienna, Währinger Gürtel 18-20, Vienna, 1090 Austria; 2 University of Vienna, Vienna, Austria; 3 Department of Laboratory Medicine, Genetics Laboratory, Medical University of Vienna, Vienna, Austria; 4 Division of Cardiology, Department of Medicine II, Medical University of Vienna, Vienna, Austria; 5 Department of Neurology, Comprehensive Center for Clinical Neurosciences and Mental Health, Medical University of Vienna, Vienna, Austria

**Keywords:** Fabry disease, Pregnancy, Pregnancy outcomes, Delivery outcomes, Pregnancy counselling, Lysosomal storage disorders

## Abstract

**Background:**

Pregnancy and delivery outcomes in women with Fabry disease are not well described.

**Methods:**

Retrospective cohort-study of women with Fabry disease in Austria using a specific questionnaire and the Austrian Mother–Child Health Passport.

**Results:**

Out of a total of 44 enrolled women (median age at study entry 44 years, p25: 30, p75: 51), 86.4% showed signs and symptoms of Fabry disease with an increase in pain burden during pregnancy, primarily in women with moderate pain before pregnancy. Thirty-two of 44 women with Fabry disease reported a total of 70 pregnancies (median age at first pregnancy 24 years, p25: 21, p75: 31), 61 (87.1%) of which resulted in 64 live births including 3 sets of twins, six miscarriages (8.6%) in five women, and three induced abortions (4.3%) in two women. Risk factors for poor maternal and foetal outcomes during pregnancy, overrepresented in our cohort as compared to the general population, were hypertension (*n* = 10, 16.4%), proteinuria (*n* = 17, 27.9%) and smoking (*n* = 24, 39.3%). Preeclampsia was reported in 7 pregnancies (11.5%). Fifty-one (79.7%) children were born at term and 13 (20.3%) were preterm (including one neonatal death), with a median gestational age of 39 weeks (p25: 38, p75: 40) and delivery by C-section in 15 pregnancies (24.6%). Thirteen (20.3%) children presented with low birth weight and 18 (28.1%) were small for their gestational age. In comparison to global and national data-sets, preeclampsia, prematurity, low birth weight, being small for their gestational age as well as inpatient stay were significantly more common in patients with Fabry disease.

**Conclusions:**

Our cohort-study in women with Fabry disease shows an increase of pain burden during pregnancies and clearly points to an increased risk for preeclampsia, prematurity, and neonates small for gestational age. With a substantial number of high-risk pregnancies, neonatal outcomes are somewhat worse in Fabry disease than in the general public. Thus, we provide valuable data enabling informed decision-making in pregnancy counselling for Fabry disease.

**Supplementary Information:**

The online version contains supplementary material available at 10.1186/s13023-024-03180-3.

## Background

Fabry disease is a rare, X-linked lysosomal storage disorder. Pathogenic variants in *GLA*, coding for alpha-galactosidase A (E.C. 3.2.1.22), lead to enzyme deficiency resulting in glycosphingolipid deposition-related sequelae in almost all organs. Quality of life and life expectancy are primarily impaired by the involvement of the heart, the kidneys and the neurological system [1].

Due to the X-linked inheritance of Fabry disease, women are more heterogeneously affected than men. A seminal study derived from the Fabry Outcome Survey (FOS) some 20 years ago clearly showed major organ involvement and clinical manifestations in female patients [2]. Thus, this hereditary, multi-systemic disease may pose health-related risks for the mother and their offspring during pregnancy, birth and the neonatal period.

So far, only a few studies have reported pregnancy outcomes in women with Fabry disease, whereas detailed delivery outcomes, apart from preterm delivery, have not been reported at all.

Bouwman and colleagues described 32 women with a history of 89 pregnancies. In comparison to a small control group, women with Fabry disease showed a higher incidence of proteinuria, whereas preeclampsia, preterm delivery, hypertension, miscarriage and intra-uterine death were not different. Notably, these outcomes were examined per woman, but not for each pregnancy [3].

In another study, Holmes and co-workers analysed 102 pregnancies among 41 women with Fabry disease. During pregnancy, the most frequent Fabry disease-related signs and symptoms comprised proteinuria, acroparesthesia, headache, constipation, and diarrhoea. Compared to the general population, there was no difference in preeclampsia, preterm delivery, hypertension, miscarriage, or intra-uterine death. However, proteinuria and gestational diabetes were more frequent among women with Fabry disease [4].

In this study, we analysed the pregnancy outcomes of women with Fabry disease in comparison to the Austrian birth registry. We further examined Fabry disease-related symptoms and changes in pain burden during pregnancies in those women. Moreover, for the first-time we have analysed the delivery outcomes of these women in detail. Thus, our data provides valuable information for pregnancy counselling in patients with Fabry disease.

## Methods

### Study design

Pregnancy outcomes of Fabry disease in Austria (PROFABIA) is a retrospective cohort-study of women with an established diagnosis of Fabry disease. We describe disease-specific symptoms as well as pregnancy and delivery outcomes in these women, whether they were diagnosed with Fabry disease at the timepoint of pregnancy or not.

### Setting and participants

We used an institutional database to identify adult women with a history of Fabry disease. This database was established in the year 2001 at the Division of Nephrology and Dialysis, Department of Medicine III, Medical University of Vienna, Austria and includes patients with variants in *GLA* referred from all over Austria.

The current study was approved by the institutional review board (IRB) at the Medical University of Vienna (unique IRB identifier: 1265/2014). All patients enrolled in the study gave written informed consent. Investigations were in accordance with the Declaration of Helsinki.

### Data sources

Demographic details as well as Fabry disease specific data, including results of genetic testing, along with the obstetrical history, including pregnancy related outcomes of children and mothers, were obtained by chart review. Data acquisition was supported by personal interviews using a specific questionnaire designed for this study, and from the Austrian Mother–Child Health Passport, which was introduced in 1974 and tracks pregnancy-related health information of the mother and child from diagnosis of pregnancy until the child’s 5th year of life [5]. Participation in this program, which is free of charge even for people without health insurance, is supported by financial incentives to the parents.

Data from the Austrian Birth Registry 2021 were obtained from "Institut für klinische Epidemiologie, Teil des Landesinstituts für Integrierte Versorgung Tirol: Geburtenregister Österreich. Bericht über die Geburtshilfe in Österreich 2021, Innsbruck" (https://www.iet.at/data.cfm?vpath=publikationen210/groe/groe-jahresbericht-2021).

For the assessments at birth of term infants, we used charts provided by Voigt et al., and for preterm born infants, we used the Fenton charts [6, 7]. For the calculation of centiles we used online calculators provided for free by Daniel Gräfe, MD (Leipzig, Germany), accessible on www.pedz.de.

### Study questionnaire

The specific questionnaire for this study addressed demographics, history, Fabry disease related signs and symptoms, as well as changes of signs and symptoms, including a pain scale, before, during, and after pregnancy and details of each pregnancy and child.

### Statistical analysis

We provide a descriptive analysis including categorical data, which are presented as count and frequency, as well as continuous variables, which are shown as median and the 25^th^ and 75^th^ percentile. We used Fisher's Exact Test for analysis of categorical data. A two-sided *p*-value < 0.05 was considered statistically significant.

## Results

### Participants

**Fig. 1:**
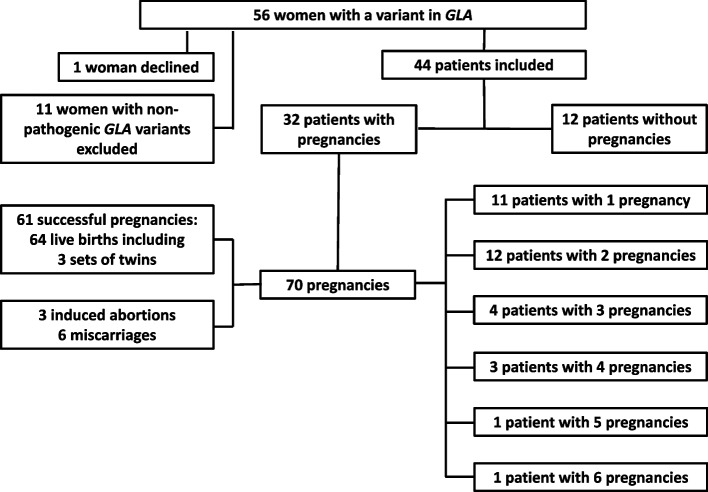
Patient disposition

As of December 2021, we identified 56 females with a variant in *GLA*, representing about 50% of women with Fabry disease in Austria and one patient refused to participate in this study. Eleven females with a non-pathogenic variant in *GLA* (p.Ser126Gly, *n* = 1; p.Ala143Thr, *n* = 3; p.Asp313Tyr, *n* = 6; c.-10C > T, *n*= 1), were excluded from the analysis [8, 9]. Twelve of the remaining 44 patients did not report any pregnancy, whereas 32 women had a history of 70 pregnancies in total (between 1971 and 2021). They gave birth to 64 children, including three sets of twins (Fig. 1).

Important characteristics of all women are summarized in Table 1. Forty-three percent received enzyme replacement or chaperone therapy. The *GLA* variants of all women are summarized in the Additional Table 1.

**Table 1:** Characteristics of 44 study participants

No. of women with Fabry disease	44
No. of families	28
Index case	15
Diagnosis by family screening	29
Ethnicity	
Caucasian	43 (97.7)
Asian	1 (2.3)
BMI, kg/m^2^	24 (22, 27)
Systolic blood pressure, mmHg	120 (110, 120)
Diastolic blood pressure, mmHg	80 (70, 80)
Hypertension, yes	9 (20.5)
Serum creatinine, mg/dl (*n* = 30)	0.8 (0.7, 0.8)
Urinary albumin to creatinine ratio, mg/g (*n* = 29)	23 (8, 59)
Urinary protein to creatinine ratio, mg/g (*n* = 29)	106 (60, 177)
Chronic kidney disease (including one kidney transplant recipient)	11 (25.0)
Age at first symptoms, years	11 (6, 20)
Age at diagnosis of Fabry disease, years	34 (21, 47)
Age at start of treatment (*n* = 21), years	46 (32, 51)
Age at study entry, years	44 (30, 51)
Fabry disease specific therapy	19 (43.2)
Current enzyme replacement therapy	16 (36.4)
Current chaperone therapy	3 (6.8)
History of pregnancies	32 (72.7)

Data are given as n (%) or median (p25, p75)

*BMI* body mass index

In 61 pregnancies, a significant proportion of women, 39.3%, reported smoking, 27.9% had proteinuria, and 16.4% were hypertensive. Preeclampsia developed in 11.5% (7/61) vs. 3.8% (39512/1039782 of European populations in the study from Abalos et al. [10], *p* = 0.0051) and 6.6% had gestational diabetes mellitus (Table 2).

**Table 2:** Characteristics at the time of pregnancy

No. of women with a successful pregnancy	32
No. of successful pregnancies	61
No. of pregnancies per woman	2 (1, 3)
Age at first pregnancy, years	24 (21, 31)
Smoking during pregnancy	24 (39.3)
Gestational diabetes mellitus	4 (6.6)
Proteinuria	17 (27.9)
Hypertension	10 (16.4)^a^
Preeclampsia	7 (11.5)
Enzyme replacement therapy during pregnancy	2 (3.3)

Data are given as n (%) or median (p25, p75)

^a^In six cases only during pregnancy

### Symptoms of Fabry disease

Fabry disease related first signs and symptoms of all women, as well as of both subgroups, with and without a history of pregnancy, are indicated in Table 3. Women who reported pregnancies appeared to have experienced first symptoms of Fabry disease earlier than women without pregnancies.

**Table 3:** First symptoms likely related to Fabry disease of 44 women

	All women (*n* = 44)	With pregnancy (*n* = 32)	Without pregnancy (*n* = 12)
Age at onset	11 (6, 20)	11 (7, 19)	14 (6, 21)
Hypohidrosis, cold or heat intolerance	4 (9.1)	4 (12.5)	0
Acroparesthesia	13 (29.5)	8 (25.0)	5 (41.7)
Pain	14 (31.8)	12 (37.5)	2 (16.7)
Angiokeratomas	1 (2.3)	1 (3.1)	0
Neurologic symptoms	5 (11.4)	5 (15.6)	0
Cerebrovascular event	2 (4.5)	2 (6.3)	0
Cardiac disease	5 (11.4)	5 (15.6)	0
Angina	0	0	0
Kidney disease	0	0	0
Proteinuria	2 (4.5)	0	2 (16.7)
Hypertension	0	0	0
Gastrointestinal symptoms	8 (18.2)	6 (18.8)	2 (16.7)
Asthma/COPD	1 (2.3)	1 (3.1)	0
Anxiety disorder	0	0	0
Depression	0	0	0
Hypacusis	0	0	0
Tinnitus	0	0	0
Vertigo	1 (2.3)	1 (3.1)	0
Cornea verticillata	2 (4.5)	1 (3.1)	1 (8.3)
Other	2 (4.5)	2 (6.3)	0

Data are given as n (%) or median (p25, p75)

*COPD* chronic obstructive pulmonary disease

Similarly, Table 4 shows all signs and symptoms of Fabry disease that occurred until participation in this study in all patients and in the subgroups with and without pregnancies.

**Table 4:** Any symptoms ever likely related to Fabry disease of 44 women

	All women (*n* = 44)	With pregnancy (*n* = 32)	Without pregnancy (*n* = 12)
Women with symptoms	38 (86.4)	28 (87.5)	10 (83.4)
Women without symptoms^a^	6 (13.7)	4 (12.5)	2 (16.7)
Age at study entry	44 (30, 51)	48 (39, 54)	24 (21, 32)
Hypohidrosis, cold or heat intolerance	21 (47.7)	15 (46.9)	6 (50.0)
Acroparesthesia	25 (56.8)	17 (53.1)	8 (66.7)
Pain	23 (52.3)	17 (53.1)	6 (50.0)
Angiokeratomas	8 (18.2)	6 (18.8)	2 (16.7)
Neurologic symptoms	10 (22.7)	9 (28.1)	1 (8.3)
Cerebrovascular event	3 (6.8)	2 (6.3)	1 (8.3)
Cardiac symptoms	15 (34.1)	14 (43.8)	1 (8.3)
Angina	8 (18.2)	8 (25.0)	0
Kidney disease	11 (25.0)	11 (34.4)	0
Proteinuria	15 (34.1)	12 (37.5)	3 (25.0)
Hypertension	12 (27.3)	12 (37.5)	0
Gastrointestinal symptoms	22 (50.0)	16 (50.0)	6 (50.0)
Asthma/COPD	11 (25.0)	11 (34.4)	0
Anxiety disorder	7 (15.9)	6 (18.8)	1 (8.3)
Depression	16 (36.4)	14 (43.8)	2 (16.7)
Hypacusis	9 (20.5)	8 (25.0)	1 (8.3)
Tinnitus	10 (22.7)	10 (31.3)	0
Vertigo	14 (31.8)	12 (37.5)	2 (16.7)
Cornea veticillata	8 (18.2)	5 (15.6)	3 (25.0)
Other	7 (15.9)	6 (18.8)	1 (8.3)

Data are given as n (%) or median (p25, p75)

*COPD* chronic obstructive pulmonary disease

^a^Including one index case with asymptomatic Fabry cardiomyopathy and five women identified by family screening

Overall, some 86% of women reported symptoms likely related to Fabry disease. Acroparesthesia, pain, and gastrointestinal symptoms were the most common disease manifestations. This did not differ between 32 women with a history of pregnancies as compared to 12 women without pregnancies. Women with a history of pregnancy were older (median age 48 years) and showed more other signs and symptoms, such as cardiac, kidney, and psychiatric symptoms, pulmonary disease, hypertension, and nervous system involvement, as compared to women without history of pregnancy (median age 24 years).

**Fig. 2:**
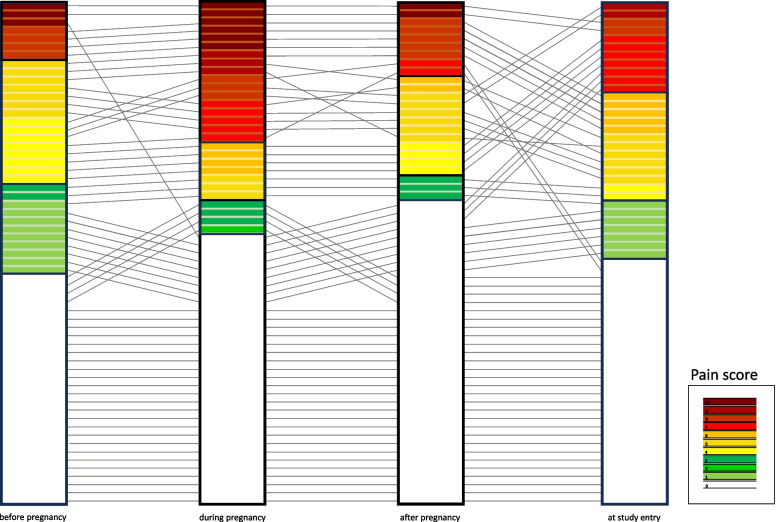
Change of pain scores and categories before, during, and following 61 pregnancies of 32 women with Fabry disease

Importantly, Fig. 2 demonstrates changes of Fabry disease related pain scores of 32 women before, during, and following 61 pregnancies up to the last follow-up for this study.

As outlined in Additional Table 2 which shows changes in pain categories, it appears that women with low pain burden before pregnancy did not show an increase in pain during pregnancy, whereas women with moderate pain before pregnancy experienced an increase in pain burden.

### Pregnancy and delivery outcomes

**Fig. 3:**
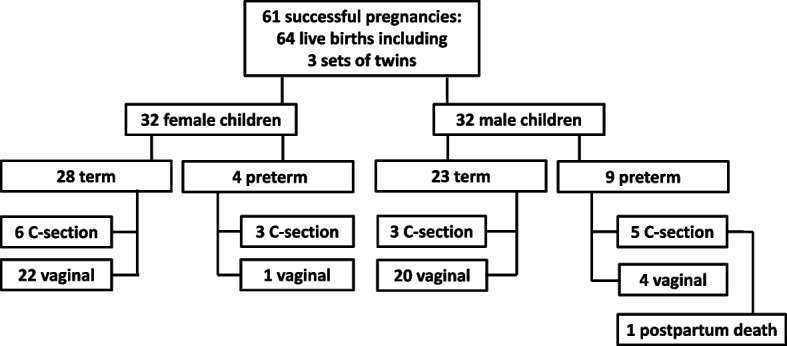
Delivery outcomes of 61 successful pregnancies in 32 women with Fabry disease

Among a total of 70 pregnancies in 32 women with Fabry disease, 61 resulted in live births of 64 children (32 female, 32 male), including 3 sets of twins (Fig. 3). Miscarriage was reported in 8.6% of pregnancies, and 4.3% were terminated by induced abortion (Table 5).

**Table 5:** Outcomes of 70 pregnancies in 32 women with Fabry disease

Outcome of pregnancy	*N* = 70
Successful pregnancies	61 (87.1)
Live birth	64^a^
Miscarriage	6 (8.6)^b^
Induced abortion	3 (4.3)^c^

Data are given as n (%)

^a^Including 3 sets of twins and 1 early neonatal death

^b^Six miscarriages in five women including one woman with two miscarriages

^c^Three induced abortions in two women

Fifty-one children were born at term and 13 were preterm. The median gestational age of all 64 children was 39 weeks and the median gestational age of preterm infants was 35 weeks (Table 6). Seventeen infants were delivered by C-section (Fig. 3).

**Table 6:** Delivery outcomes of 61 successful pregnancies

	All	Female children	Male children
Women	32	23	21
Number of pregnancies	61	31	31
Mothers age at delivery, years	28 (23, 33)	30 (25, 34)	26 (23, 32)
Pregnancy terminated by C-section	15 (24.6)	8 (25.8)	8 (25.8)
Infants	64^a^^,b^	32^a^	32^b^^,c^
Gestational age, weeks	39 (38, 40)	40 (38, 40)	39 (36, 40)
Preterm infants	13 (20.3)	4 (12.5)	9 (28.1)
Gestational age of preterm infants, weeks	35 (34, 36)	35 (33, 35)	35 (34, 36)
Weight at birth, g	3020 (2608, 3419)	2900 (2528, 3400)	3115 (2650, 3463)
Weight, centile	23 (8, 49)	21 (6, 58)	34 (12, 48)
Missing	2 (3.1)	1 (3.1)	1 (3.1)
Low birth weight	13 (20.3)	7 (21.9)	6 (18.8)
SGA	18 (28.1)	11 (34.4)	7 (21.9)
Length at birth, cm	49 (47,51)	49 (46, 51)	50 (47, 51)
Length, centile	20 (4, 46)	21 (3, 53)	19 (4, 35)
Missing	4 (6.3)	1 (3.1)	3 (9.4)
Head circumference, cm	34 (32, 35)	33 (31, 35)	34 (34, 35)
Head, centile	32 (13, 62)	29 (6, 49)	41 (17, 71)
Missing	23 (35.9)	13 (40.6)	10 (31.3)
Apgar 1	9 (9, 9)	9 (9, 9)	9 (9, 9)
Missing	8 (12.5)	4 (12.5)	4 (12.5)
Apgar 5	10 (10, 10)	10 (10, 10)	10 (10, 10)
Missing	8 (12.5)	4 (12.5)	4 (12.5)
Apgar 10	10 (10, 10)	10 (10, 10)	10 (10,10)
Missing	8 (12.5)	4 (12.5)	4 (12.5)
Malformations	11 (17.2)	5 (15.6)	6 (18.8)
Missing	1 (1.6)	0	1 (3.1)
Inpatient stay	19 (29.7)	8 (25.0)	11 (34.4)

Data are given as n (%) or median (p25, p75)

*SGA* small for gestational age

^a^3 females from 2 sets of twins

^b^3 males from 2 sets of twins

^c^Including one child that died two days after delivery

Eighteen children were small for gestational age and 11 showed malformations. Details of length, weight, and head circumference at birth, including the corresponding centiles, as well as APGAR scores and inpatient stay, are indicated in Table 6 for the group as a whole and for female and male infants.

In comparison to the Austrian birth registry from 2021 (Table 7), children born to mothers with Fabry disease were more likely to be preterm (*p* = 0.0006), had a lower birth weight (*p* = 0.0001), and were small for their gestational age more than twice as often (*p* < 0.0001). Inpatient stay of the neonate was also more frequent in Fabry disease (*p* < 0.0001).

**Table 7:** Comparison of delivery outcomes from 61 pregnancies among 32 women with Fabry disease and the general population in Austria

	Women with Fabry disease (1971–2021)	Birth registry Austria (2021)	*P*
Women with live births	32	83,721	
Live births	64^a^	84,989	
Female neonates	32/64 (50.0)	41,332/84983 (48.6)	0.9
C-section	17/64 (26.6)^b^	27,058/84981 (31.8)	0.42
Preterm	13/64 (20.3)	6195/84968 (7.3)	**0.0006**
Low birth weight	13/64 (20.3)	5118/84890 (6.2)	**0.0001**
Small for gestational age	18/64 (28.1)	7887/84847 (9.3)	** < 0.0001**
Inpatient stay	18/64 (28.1)	6666/84989 (7.8)	** < 0.0001**
Twin pregnancies	3/61 (4.9)	1253/84018 (1.5)	0.06
Post-partum death	1/64 (1.6)	155/84834 (1.8 ‰)	0.11

Data are given as n/total count (%)

^a^Including three sets of twins

^b^Including two sets of twins delivered by C-section

The Additional Fig. 1 shows the results of genetic testing, as well as Fabry disease-specific therapy of 32 female and 32 male children. The median age of these descendants was 21.4 (p25: 13.1; p75: 28.3) years as of December 2021. Overall, 8 children were not tested for the presence of a *GLA* variant and 24 showed no inherited variant in *GLA*. Among 32 children with a variant in *GLA,* 11 received a Fabry disease specific therapy during the observational period.

## Discussion

Our study of pregnancy and delivery outcomes among women with Fabry disease showed an increase of pain burden during pregnancy in women with moderate pain before pregnancy. The risk for miscarriage was not increased in Fabry disease. Preeclampsia occurred more frequently and neonates were more often preterm, had a lower birth weight, and were more commonly small for gestational age as compared to global data and the general population in Austria, respectively.

In a recent study from Spain, 83.5% of women showed signs and symptoms of Fabry disease, which compares well to the present cohort of 44 women of whom 86.4% reported Fabry disease related symptoms [11]. In our study, acroparesthesia, pain, and gastrointestinal symptoms were the most common disease manifestations. This did not differ between 32 women with a history of pregnancies as compared to 12 women without pregnancies. However, women with a history of pregnancy were older and presented more frequently with other signs and symptoms as outlined in Table 4. Thus, age is likely associated with both, the symptoms and the likelihood of having had a pregnancy.

Forty-three percent of all 44 women in our study received Fabry disease therapy with enzyme replacement or a chaperone. This observation is very similar to the 34% of treated patients in the Spanish study and analogical points to a shortfall of specific therapy in women with Fabry disease [11]. Only two patients received enzyme replacement therapy during pregnancy in our study. Regarding treatment during pregnancy the SmPC of agalsidase beta points out that agalsidase beta must not be used during pregnancy unless clearly necessary. In case of agalsidase alfa the SmPC mentions that only very limited data are available on agalsidase alfa-exposed pregnant women. For the use during pregnancy caution is advised. There is no clear recommendation for treatment during pregnancy with enzyme replacement therapy. At this point, it is worth mentioning that chaperone therapy is not licensed for use in pregnancy [12].

Only one previous study reported Fabry disease-related symptoms during pregnancy. Among these, acroparesthesia was reported in 31.3% and headaches in 22.5% [4]. However, changes in pain burden during pregnancy were not ascertained in that study. In contrast, our study employed a pain scale that clearly showed an increase in pain burden during pregnancy, primarily in women with moderate pain before pregnancy, whereas women with no or low pain burden before pregnancy did not experience a substantial change in pain during pregnancy.

The main maternal risk factors for poor pregnancy, delivery, and neonatal outcomes, still overrepresented in our cohort as compared to the general population, were hypertension, signs of chronic kidney disease, and smoking. These findings compare well with other cohorts of women with Fabry disease bearing children [3, 4].

Importantly, preeclampsia is a life-threatening disease of pregnancy and plays a decisive role in maternal and neonatal morbidity and mortality [13]. From a global perspective, the regional rates of preeclampsia vary between 1% and 5.6%, with 3.8% in the European region [10]. In our study, as well as in two other studies of Fabry disease, the prevalence of preeclampsia was 11.5% and 4.9% or 9.4%, respectively [3, 4]. Thus, the aforementioned studies emphasize a substantially increased risk for preeclampsia in Fabry disease.

Among pregnancy outcomes, the rate of successful pregnancies resulting in a live birth was 87.1% and the women enrolled in our study reported three induced abortions. Furthermore, the rate of miscarriages, 8.6% (*n* = 6), was similar to the study of Holmes and colleagues (11.8%) [4]. Contrary to the aforementioned studies, Bouwman et al. observed miscarriages in 25% of participants (not pregnancies) of their study. Through this, the rate of miscarriages of all pregnancies in this study remains elusive [3]. Notwithstanding, a recent study of the general population in Norway showed a miscarriage risk of around 10% in women aged 25–29, which rises rapidly after age 30, reaching 53% in women aged 45 and over [14]. Thus, we did not observe an overall increased risk of miscarriages in women with Fabry disease.

This first report on detailed delivery outcomes of pregnancies in women with Fabry disease disclosed a higher rate of preterm infants (20.3%) as compared to the Austrian population (7.3%). This is in line with the data provided by Holmes (18.5% of pregnancies) and Bouwman (19% of women) [3, 4]. In this context, a recent systematic review showed a preterm birth rate of 7.9% in developed countries in the year 2020 [15]. By way of contrast, the preterm birth rate in this analysis was highest in southern Asia (13.2%) [15]. Thus, prematurity in Fabry disease is even more frequent as compared to less developed countries and compares more or less to other hereditary disease such as complement mediated thrombotic microangiopathy [16].

The rate of C-sections was not different in women with Fabry disease as compared to the general population in Austria. Weight at birth of female and male infants was lower, with 20.3% of infants presenting with low birth weight and 28.1% of infants being small for gestational age compared to 9.3% of the Austrian population. Thus, it is tempting to speculate that Fabry disease manifestations of the mother, together with the consequences of glycosphingolipid deposits within multiple cell types of the placenta, cord, and membranes, are the culprits of the somewhat worse neonatal outcomes in Fabry disease [17].

Nevertheless, neonatal outcomes in our study were satisfactory. Follow-up of the offspring within this study showed 57.1% of genetically tested children harbouring an inherited variant in *GLA*. Among daughters and sons with Fabry disease, 29.4% (5 of 17) and 46.7% (7 of 15) received Fabry disease specific therapy by the end of this study.

The strengths of our study comprise the completeness of pregnancy and neonatal data accomplished by the Austrian Mother–Child Health Passport. Furthermore, data was also collected by structured personal interviews supported by a study-specific questionnaire. Of course, a recall bias regarding miscarriages and signs and symptoms of Fabry disease in previous pregnancies cannot be ruled out. Although the sample size is small, the results of this study in a rare disease should be considered generalizable to the population of patients with Fabry disease living in regions with advanced healthcare.

## Conclusions

In conclusion, our study in women with Fabry disease shows an increase of pain burden during pregnancies in those with pre-existing moderate pain and clearly points to an increased risk for preeclampsia, for prematurity, and for neonates small for gestational age. Despite a substantial number of high-risk pregnancies, neonatal outcomes were acceptable in this study on Fabry disease. Thus, we provide a valuable contribution to help enable an informed discussion around pregnancy and pregnancy outcomes in Fabry disease.

### Supplementary Information

**Additional file 1:** **Additional Figure 1.** Results of genetic testing and Fabry specific therapy of children born to mothers with Fabry disease (positive: variant in *GLA*; negative: no variant in *GLA*).

**Additional file 2:** **Additional Table 1.** *GLA* (NM_000169.3) variants of 44 study participants from 28 families.

**Additional file 3:** **Additional Table 2.** Pain categories during pregnancy, post pregnancy, and at study entry as compared to pain before pregnancy in 32 women and 61 pregnancies.

**Additional file 4:** **Additional Spreadsheet 1.** Individual data of women with Fabry disease.

**Additional file 5:** **Additional Spreadsheet 2**. Individual data of children born to mothers with Fabry disease.

## Data Availability

The datasets used for this study are available as additional supporting files: Additional Spreadsheet 1. Individual data of women with Fabry disease, and Additional Spreadsheet 2. Individual data of children born to mothers with Fabry disease.
