# Supplementary material for: Pregnancy outcomes of Fabry disease in Austria (PROFABIA)-a retrospective cohort-study
Source: Orphanet J Rare Dis. 2024 Apr 18;19:165. doi: 10.1186/s13023-024-03180-3 (PMC11025160; doi:10.1186/s13023-024-03180-3)
Supplement: Supplementary file 1 — Additional file 1: Additional Figure 1. Results of genetic testing and Fabry specific therapy of children born to mothers with Fabry disease (positive: variant in GLA; negative: no variant in GLA). [file 13023_2024_3180_MOESM1_ESM.pptx]

## Slide 1
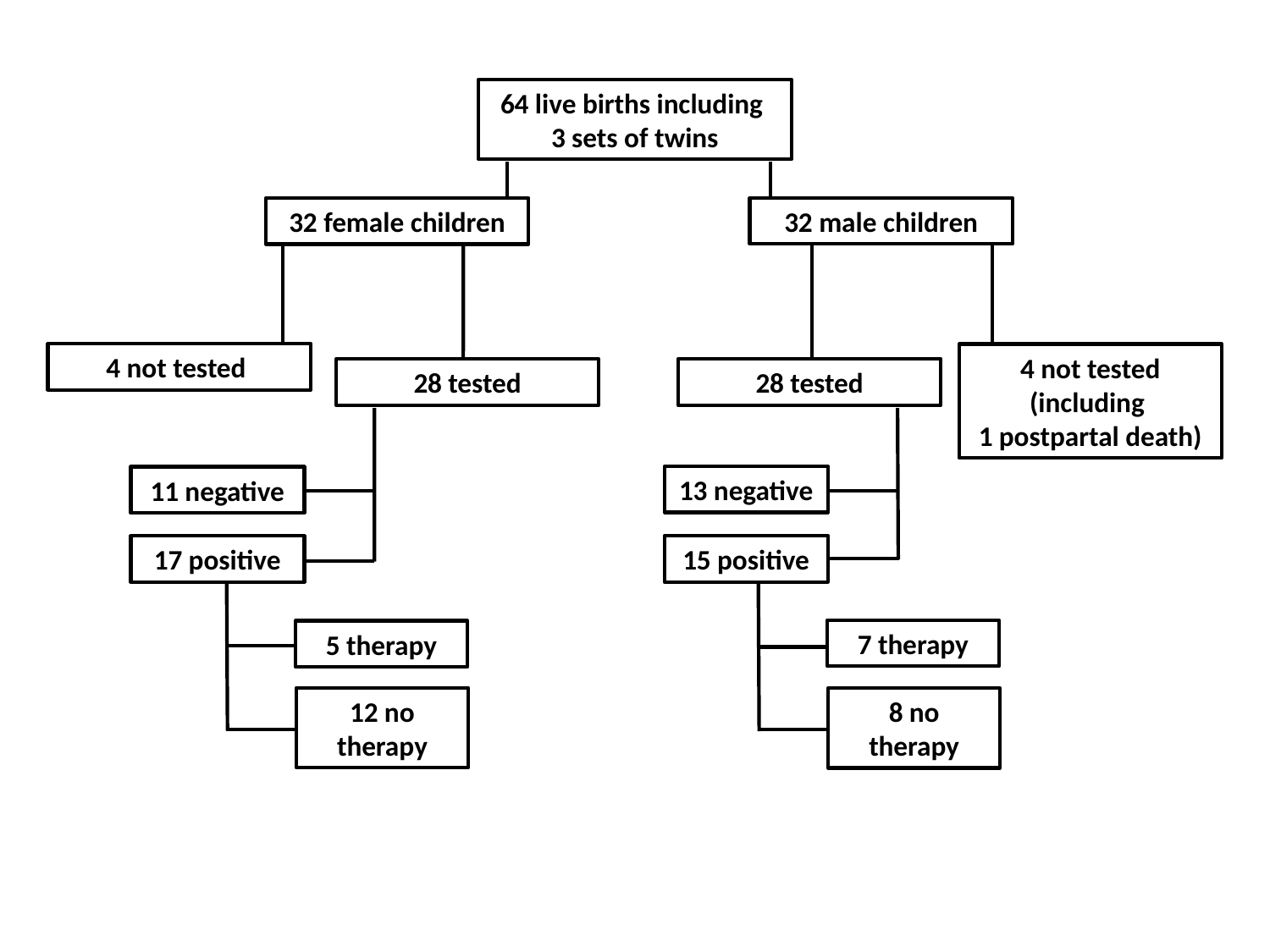

64 live births including
3 sets of twins
32 male children
32 female children
4 not tested
4 not tested (including
1 postpartal death)
28 tested
28 tested
13 negative
11 negative
15 positive
17 positive
7 therapy
5 therapy
12 no therapy
8 no therapy
