# Supplementary material for: Pregnancy outcomes of Fabry disease in Austria (PROFABIA)-a retrospective cohort-study
Source: Orphanet J Rare Dis. 2024 Apr 18;19:165. doi: 10.1186/s13023-024-03180-3 (PMC11025160; doi:10.1186/s13023-024-03180-3)
Supplement: Supplementary file 2 — Additional file 2: Additional Table 1. GLA (NM_000169.3) variants of 44 study participants from 28 families. [file 13023_2024_3180_MOESM2_ESM.docx]

**Additional Table 1.** *GLA* (NM_000169.3) variants of 44 study participants from 28 families.

*GLA* variant Count Families

| c.59C>A, p.(Ala20Asp) | 3 | 1 |
| --- | --- | --- |
| c.103G>A, p.(Gly35Arg) | 1 | 1 |
| c.169C>T, p.(Gln57*) | 1 | 1 |
| c.319C>T, p.(Gln107*) | 1 | 1 |
| c.335G>A, p.(Arg112His) | 11 | 5 |
| c.568delG, p.(Ala190Profs*2) | 3 | 1 |
| c.644A>G, p.(Asn215Ser) | 1 | 1 |
| c.675_694del20bp, p.(Trp226*) | 1 | 1 |
| c.716T>C, p.(Ile239Thr) | 3 | 1 |
| c.772G>A, p.(Gly258Arg) | 1 | 1 |
| c.800T>C, p.(Met267Thr) | 2 | 1 |
| c.850A>G, p.(Met284Val) | 1 | 1 |
| c.875C>T, p.(Ala292Val) | 1 | 1 |
| c.902G>A, p.(Arg301Gln) | 2 | 2 |
| c.947T>G, p.(Val316Gly) | 1 | 1 |
| c.958A>T, p.(Asn320Tyr) | 2 | 1 |
| c.997C>T, p.(Gln333T*) | 1 | 1 |
| c.1010T>C, p.(Phe337Ser) | 1 | 1 |
| c.1025G>A, p.(Arg342Gln) | 1 | 1 |
| c.1132T>C, p.(Cys378Arg) | 3 | 1 |
| c.1272_1274delATTinsAluYb8, p.(Leu425Alafs*29) | 1 | 1 |
| c.1288T>C, p.(*430Glnext*30) | 1 | 1 |
| c.547+1G>C | 1 | 1 |
