# Supplementary material for: Pregnancy outcomes of Fabry disease in Austria (PROFABIA)-a retrospective cohort-study
Source: Orphanet J Rare Dis. 2024 Apr 18;19:165. doi: 10.1186/s13023-024-03180-3 (PMC11025160; doi:10.1186/s13023-024-03180-3)
Supplement: Supplementary file 3 — Additional file 3: Additional Table 2. Pain categories during pregnancy, post pregnancy, and at study entry as compared to pain before pregnancy in 32 women and 61 pregnancies. [file 13023_2024_3180_MOESM3_ESM.docx]

**Additional Table 2.** Pain categories during pregnancy, post pregnancy, and at study entry as compared to pain before pregnancy in 32 women and 61 pregnancies.

| Pain before pregnancy, n=61 | Pain during pregnancy  No Low Moderate Severe | | | |
| --- | --- | --- | --- | --- |
| O (n=28) | n=24 | n=4 | n=0 | n=0 |
| Low (n=11) | n=8 | n=0 | n=3 | n=0 |
| Moderate (n=15) | n=0 | n=0 | n=4 | n=11 |
| Severe (n=7) | n=1 | n=0 | n=0 | n=6 |

| Pain before pregnancy, n=61 | Pain post pregnancy  No Low Moderate Severe | | | |
| --- | --- | --- | --- | --- |
| 0 (n=28) | n=28 | n=0 | n=0 | n=0 |
| Low (n=11) | n=8 | n=3 | n=0 | n=0 |
| Moderate (n=15) | n=0 | n=0 | n=12 | n=3 |
| Severe (n=7) | n=1 | n=0 | n=0 | n=6 |

| Pain before pregnancy, n=61 | Pain at study entry  No Low Moderate Severe | | | |
| --- | --- | --- | --- | --- |
| 0 (n=28) | n=28 | n=0 | n=0 | n=0 |
| Low (n=11) | n=0 | n=7 | n=2 | n=2 |
| Moderate (n=15) | n=2 | n=0 | n=7 | n=6 |
| Severe (n=7) | n=0 | n=0 | n=4 | n=3 |

Pain categories according to pain scores: 0 = no pain, 1-3 = low pain, 4-6 = moderate pain, 7-10 = severe pain
